# Supplementary material for: Deletion of wfs1 Impairs Oligodendrocyte Precursor Cells Dorsal Distribution and Myelination Through the wfs1-hmgcs1 Axis in Zebrafish
Source: Biology (Basel). 2026 Aug 16;15(16):1402. doi: 10.3390/biology15161402 (PMC13510151; doi:10.3390/biology15161402)
Supplement: Supplementary file 1 [file biology-15-01402-s001.zip › File S1-Transcriptome sequencing data and differentially expressed genes (DEGs) in wfs1-- zebrafish at 5 dpf/1.GO/wfs1KOvsWT/GSEA/WT/CELL_CORTEX/CELL_CORTEX(GO_0005938).html]

Details for gene set CELL\_CORTEX(GO:0005938)[GSEA]


|  || Dataset | deg.sample |
| Phenotype | sample.cls |
| Upregulated in class | WT |
| GeneSet | CELL\_CORTEX(GO:0005938) |
| Enrichment Score (ES) | -0.3610522 |
| Normalized Enrichment Score (NES) | -1.7901515 |
| Nominal p-value | 0.0 |
| FDR q-value | 0.28328174 |
| FWER p-Value | 0.707 |
Table: GSEA Results Summary

  

Fig 1: Enrichment plot: CELL\_CORTEX(GO:0005938)
  
Profile of the Running ES Score & Positions of GeneSet Members on the Rank Ordered List

  

| PROBE | DESCRIPTION   (from dataset) | GENE SYMBOL | GENE\_TITLE | RANK IN GENE LIST | RANK METRIC SCORE | RUNNING ES | CORE ENRICHMENT || 1 | 436718 | rhocb |  |  | 948 | 2.171 | -0.0039 | No |
| 2 | 641575 | actn2b |  |  | 1758 | 1.499 | -0.0114 | No |
| 3 | 553415 | rhobtb2a |  |  | 3000 | 1.035 | -0.0382 | No |
| 4 | 572002 | erc2 |  |  | 3148 | 1.002 | -0.0308 | No |
| 5 | 562804 | si:dkey-6b12.5 |  |  | 4441 | 0.754 | -0.0625 | No |
| 6 | 792949 | fryb |  |  | 4977 | 0.685 | -0.0712 | No |
| 7 | 100002992 | apnl |  |  | 5004 | 0.680 | -0.0638 | No |
| 8 | 100124652 | pard6b |  |  | 5488 | 0.635 | -0.0715 | No |
| 9 | 557015 | mlphb |  |  | 5669 | 0.620 | -0.0697 | No |
| 10 | 563414 | rims3 |  |  | 5917 | 0.595 | -0.0704 | No |
| 11 | 30724 | melk |  |  | 5996 | 0.587 | -0.0658 | No |
| 12 | 101882577 | si:ch211-13f8.1 |  |  | 6067 | 0.580 | -0.0611 | No |
| 13 | 30741 | ctnna1 |  |  | 6114 | 0.575 | -0.0556 | No |
| 14 | 566840 | si:dkey-121a11.3 |  |  | 6167 | 0.571 | -0.0504 | No |
| 15 | 556486 | sept4a |  |  | 6511 | 0.536 | -0.0548 | No |
| 16 | 80963 | rnd3a |  |  | 6599 | 0.528 | -0.0512 | No |
| 17 | 560668 | phldb2b |  |  | 6634 | 0.525 | -0.0460 | No |
| 18 | 335987 | gypc |  |  | 6703 | 0.519 | -0.0420 | No |
| 19 | 751645 | rnd2 |  |  | 6720 | 0.517 | -0.0363 | No |
| 20 | 568029 | exoc8 |  |  | 7083 | 0.485 | -0.0419 | No |
| 21 | 445406 | ect2 |  |  | 7185 | 0.475 | -0.0394 | No |
| 22 | 558173 | fryl |  |  | 7323 | 0.465 | -0.0381 | No |
| 23 | 80959 | pard6gb |  |  | 7495 | 0.454 | -0.0381 | No |
| 24 | 100006132 | si:ch211-1f22.2 |  |  | 8076 | 0.417 | -0.0514 | No |
| 25 | 553729 | dbnlb |  |  | 8709 | 0.387 | -0.0667 | No |
| 26 | 394136 | sept7a |  |  | 9042 | 0.365 | -0.0728 | No |
| 27 | 399546 | pxna |  |  | 9208 | 0.353 | -0.0737 | No |
| 28 | 570807 | phldb2a |  |  | 9558 | 0.330 | -0.0808 | No |
| 29 | 336933 | rhogb |  |  | 9858 | 0.312 | -0.0865 | No |
| 30 | 571702 | sept8b |  |  | 9890 | 0.310 | -0.0837 | No |
| 31 | 795670 | llgl2 |  |  | 9936 | 0.307 | -0.0815 | No |
| 32 | 569366 | tnfaip2b |  |  | 10038 | 0.301 | -0.0811 | No |
| 33 | 573655 | rhof |  |  | 10295 | 0.285 | -0.0857 | No |
| 34 | 560400 | actn1 |  |  | 10299 | 0.285 | -0.0824 | No |
| 35 | 568203 | arv1 |  |  | 10861 | 0.251 | -0.0971 | No |
| 36 | 58026 | psen2 |  |  | 11048 | 0.241 | -0.1001 | No |
| 37 | 393672 | rims2a |  |  | 11127 | 0.238 | -0.0997 | No |
| 38 | 556424 | rhoga |  |  | 11444 | 0.220 | -0.1070 | No |
| 39 | 30221 | psen1 |  |  | 11456 | 0.219 | -0.1047 | No |
| 40 | 558920 | amotl2a |  |  | 11469 | 0.218 | -0.1025 | No |
| 41 | 322338 | exoc1 |  |  | 11688 | 0.207 | -0.1069 | No |
| 42 | 556873 | si:ch211-175f11.5 |  |  | 11753 | 0.203 | -0.1065 | No |
| 43 | 559035 | shroom4 |  |  | 11768 | 0.202 | -0.1045 | No |
| 44 | 447899 | rtkn2b |  |  | 11830 | 0.199 | -0.1040 | No |
| 45 | 562517 | anln |  |  | 12145 | 0.182 | -0.1118 | No |
| 46 | 561153 | dbn1 |  |  | 12276 | 0.175 | -0.1138 | No |
| 47 | 321155 | si:dkey-238i5.2 |  |  | 12314 | 0.173 | -0.1129 | No |
| 48 | 445325 | sept5b |  |  | 12645 | 0.157 | -0.1214 | No |
| 49 | 30131 | dla |  |  | 12964 | 0.142 | -0.1297 | No |
| 50 | 559010 | exoc5 |  |  | 13041 | 0.138 | -0.1304 | No |
| 51 | 572344 | numa1 |  |  | 13264 | 0.127 | -0.1359 | No |
| 52 | 100149472 | pard3ba |  |  | 13297 | 0.125 | -0.1354 | No |
| 53 | 559364 | shroom1 |  |  | 13454 | 0.118 | -0.1389 | No |
| 54 | 101180909 | shroom3 |  |  | 14104 | 0.087 | -0.1583 | No |
| 55 | 559866 | exoc4 |  |  | 14147 | 0.085 | -0.1586 | No |
| 56 | 100170824 | pard6ga |  |  | 14339 | 0.077 | -0.1638 | No |
| 57 | 373082 | gys2 |  |  | 14567 | 0.066 | -0.1701 | No |
| 58 | 562077 | clasp1a |  |  | 14726 | 0.059 | -0.1744 | No |
| 59 | 323457 | sept2 |  |  | 14860 | 0.053 | -0.1779 | No |
| 60 | 403050 | pard3ab |  |  | 15104 | 0.041 | -0.1851 | No |
| 61 | 100149357 | rims1a |  |  | 15345 | 0.030 | -0.1923 | No |
| 62 | 562190 | macf1a |  |  | 15397 | 0.027 | -0.1936 | No |
| 63 | 114424 | cdh1 |  |  | 15458 | 0.024 | -0.1952 | No |
| 64 | 335925 | sept5a |  |  | 15509 | 0.022 | -0.1965 | No |
| 65 | 317741 | coro1ca |  |  | 15805 | 0.006 | -0.2057 | No |
| 66 | 568486 | LOC568486 |  |  | 15988 | -0.001 | -0.2115 | No |
| 67 | 553413 | rhoh |  |  | 16095 | -0.005 | -0.2147 | No |
| 68 | 569434 | arhgap32b |  |  | 16297 | -0.011 | -0.2209 | No |
| 69 | 80972 | dvl3a |  |  | 16463 | -0.016 | -0.2259 | No |
| 70 | 566287 | phldb1a |  |  | 17082 | -0.036 | -0.2450 | No |
| 71 | 564545 | si:dkey-40c11.1 |  |  | 17126 | -0.037 | -0.2459 | No |
| 72 | 795167 | rac3b |  |  | 17171 | -0.040 | -0.2468 | No |
| 73 | 101882842 | si:dkey-45k15.1 |  |  | 17377 | -0.049 | -0.2527 | No |
| 74 | 406318 | clta |  |  | 17484 | -0.053 | -0.2554 | No |
| 75 | 406591 | exoc6 |  |  | 17492 | -0.053 | -0.2550 | No |
| 76 | 394125 | rhoca |  |  | 17514 | -0.054 | -0.2550 | No |
| 77 | 751760 | llgl1 |  |  | 17716 | -0.063 | -0.2606 | No |
| 78 | 323216 | lasp1 |  |  | 17758 | -0.065 | -0.2611 | No |
| 79 | 100034367 | utrn |  |  | 17796 | -0.067 | -0.2614 | No |
| 80 | 445240 | fnbp1l |  |  | 18124 | -0.082 | -0.2708 | No |
| 81 | 565842 | pclob |  |  | 18142 | -0.083 | -0.2703 | No |
| 82 | 550129 | sept10 |  |  | 18262 | -0.088 | -0.2730 | No |
| 83 | 406411 | rhoaa |  |  | 18330 | -0.091 | -0.2740 | No |
| 84 | 324681 | rhogc |  |  | 18482 | -0.098 | -0.2776 | No |
| 85 | 30265 | ctnnb1 |  |  | 18549 | -0.101 | -0.2785 | No |
| 86 | 570923 | rims4 |  |  | 18921 | -0.119 | -0.2888 | No |
| 87 | 792631 | si:dkey-263f15.2 |  |  | 18924 | -0.119 | -0.2874 | No |
| 88 | 321590 | sept8a |  |  | 19001 | -0.121 | -0.2884 | No |
| 89 | 327204 | rac1a |  |  | 19053 | -0.124 | -0.2885 | No |
| 90 | 431748 | dbnla |  |  | 19153 | -0.129 | -0.2901 | No |
| 91 | 567832 | exoc3l1 |  |  | 19164 | -0.130 | -0.2888 | No |
| 92 | 323630 | dvl2 |  |  | 19187 | -0.130 | -0.2880 | No |
| 93 | 554123 | sept3 |  |  | 19212 | -0.131 | -0.2871 | No |
| 94 | 566822 | frya |  |  | 19310 | -0.136 | -0.2886 | No |
| 95 | 553383 | spna2 |  |  | 19315 | -0.136 | -0.2871 | No |
| 96 | 30615 | acvr1l |  |  | 19442 | -0.141 | -0.2894 | No |
| 97 | 100002110 | stxbp6l |  |  | 19526 | -0.145 | -0.2902 | No |
| 98 | 404604 | clasp2 |  |  | 19579 | -0.147 | -0.2901 | No |
| 99 | 563393 | rims2b |  |  | 19595 | -0.148 | -0.2888 | No |
| 100 | 100000597 | sept7b |  |  | 20105 | -0.171 | -0.3028 | No |
| 101 | 336381 | cotl1 |  |  | 20136 | -0.172 | -0.3017 | No |
| 102 | 100006671 | zgc:165653 |  |  | 20161 | -0.173 | -0.3004 | No |
| 103 | 553051 | fermt2 |  |  | 20702 | -0.199 | -0.3150 | No |
| 104 | 393431 | zgc:63587 |  |  | 20871 | -0.208 | -0.3178 | No |
| 105 | 337243 | sept9a |  |  | 21278 | -0.229 | -0.3279 | No |
| 106 | 553451 | sptbn1 |  |  | 21336 | -0.233 | -0.3269 | No |
| 107 | 393653 | rhogd |  |  | 21640 | -0.248 | -0.3335 | No |
| 108 | 108179043 | LOC108179043 |  |  | 21746 | -0.254 | -0.3338 | No |
| 109 | 564600 | sept12 |  |  | 22004 | -0.267 | -0.3387 | No |
| 110 | 559643 | epb41a |  |  | 22068 | -0.270 | -0.3374 | No |
| 111 | 562146 | pard3bb |  |  | 22183 | -0.277 | -0.3377 | No |
| 112 | 562449 | arhgap32a |  |  | 22284 | -0.283 | -0.3374 | No |
| 113 | 559353 | si:dkey-30c15.10 |  |  | 22400 | -0.290 | -0.3376 | No |
| 114 | 561905 | mlpha |  |  | 22511 | -0.297 | -0.3375 | No |
| 115 | 492470 | spire2 |  |  | 22636 | -0.304 | -0.3378 | No |
| 116 | 570275 | phldb1b |  |  | 23099 | -0.334 | -0.3483 | No |
| 117 | 492762 | trip10a |  |  | 23151 | -0.337 | -0.3459 | No |
| 118 | 406659 | rhoac |  |  | 23154 | -0.338 | -0.3419 | No |
| 119 | 563246 | exoc3l4 |  |  | 23228 | -0.343 | -0.3401 | No |
| 120 | 553301 | exoc3l2a |  |  | 23290 | -0.347 | -0.3379 | No |
| 121 | 322694 | sept6 |  |  | 23764 | -0.378 | -0.3483 | No |
| 122 | 563056 | hcls1 |  |  | 23829 | -0.382 | -0.3457 | No |
| 123 | 323320 | actn3b |  |  | 23907 | -0.386 | -0.3435 | No |
| 124 | 58040 | sptb |  |  | 23985 | -0.391 | -0.3413 | No |
| 125 | 563576 | si:dkey-245p14.4 |  |  | 24471 | -0.418 | -0.3516 | No |
| 126 | 100006041 | rhoab |  |  | 24773 | -0.436 | -0.3558 | Yes |
| 127 | 100148002 | pcloa |  |  | 24935 | -0.443 | -0.3556 | Yes |
| 128 | 558037 | zgc:162239 |  |  | 25031 | -0.449 | -0.3532 | Yes |
| 129 | 83773 | dmd |  |  | 25168 | -0.457 | -0.3520 | Yes |
| 130 | 406723 | exoc7 |  |  | 25188 | -0.459 | -0.3471 | Yes |
| 131 | 561671 | si:ch211-106h4.9 |  |  | 25313 | -0.468 | -0.3455 | Yes |
| 132 | 100001110 | org |  |  | 25776 | -0.509 | -0.3539 | Yes |
| 133 | 405780 | rnd1a |  |  | 25857 | -0.516 | -0.3503 | Yes |
| 134 | 567159 | tnfaip2a |  |  | 26029 | -0.532 | -0.3493 | Yes |
| 135 | 569174 | zgc:114123 |  |  | 26088 | -0.537 | -0.3447 | Yes |
| 136 | 101883199 | si:dkey-196h17.9 |  |  | 26324 | -0.559 | -0.3454 | Yes |
| 137 | 437027 | rac3a |  |  | 26579 | -0.586 | -0.3464 | Yes |
| 138 | 798260 | si:dkey-40c11.2 |  |  | 26679 | -0.596 | -0.3424 | Yes |
| 139 | 767763 | exoc6b |  |  | 26763 | -0.605 | -0.3378 | Yes |
| 140 | 565844 | rhobtb2b |  |  | 26936 | -0.623 | -0.3357 | Yes |
| 141 | 415151 | rac2 |  |  | 27490 | -0.672 | -0.3451 | Yes |
| 142 | 436864 | rnd3b |  |  | 27850 | -0.722 | -0.3478 | Yes |
| 143 | 562838 | rac1b |  |  | 28146 | -0.763 | -0.3480 | Yes |
| 144 | 100003123 | si:dkey-27p18.5 |  |  | 28509 | -0.823 | -0.3495 | Yes |
| 145 | 569891 | nsmfb |  |  | 28573 | -0.835 | -0.3415 | Yes |
| 146 | 565130 | pxnb |  |  | 28810 | -0.880 | -0.3384 | Yes |
| 147 | 100006166 | si:ch211-1f22.1 |  |  | 28986 | -0.917 | -0.3329 | Yes |
| 148 | 326287 | epb41b |  |  | 29168 | -0.963 | -0.3271 | Yes |
| 149 | 792699 | rac1l |  |  | 30004 | -1.247 | -0.3385 | Yes |
| 150 | 560003 | erc1b |  |  | 30222 | -1.351 | -0.3292 | Yes |
| 151 | 565839 | exoc3l2b |  |  | 30237 | -1.358 | -0.3134 | Yes |
| 152 | 555837 | cttnbp2 |  |  | 30631 | -1.614 | -0.3064 | Yes |
| 153 | 100004870 | exoc2 |  |  | 30650 | -1.633 | -0.2875 | Yes |
| 154 | 548606 | erc1a |  |  | 30852 | -1.813 | -0.2721 | Yes |
| 155 | 557962 | spire1a |  |  | 30915 | -1.880 | -0.2515 | Yes |
| 156 | 58037 | actn3a |  |  | 30981 | -1.946 | -0.2303 | Yes |
| 157 | 100005049 | fgf1b |  |  | 31129 | -2.123 | -0.2095 | Yes |
| 158 | 792259 | sept15 |  |  | 31355 | -2.543 | -0.1862 | Yes |
| 159 | 334015 | exoc3 |  |  | 31457 | -2.792 | -0.1559 | Yes |
| 160 | 557239 | antxr2a |  |  | 31582 | -3.209 | -0.1214 | Yes |
| 161 | 553414 | rnd1b |  |  | 31592 | -3.235 | -0.0830 | Yes |
| 162 | 541494 | sept9b |  |  | 31689 | -3.641 | -0.0424 | Yes |
| 163 | 792828 | pard6a |  |  | 31747 | -4.071 | 0.0045 | Yes |
Table: GSEA details
[plain text format]

  

Fig 2: CELL\_CORTEX(GO:0005938)
  
Blue-Pink O' Gram in the Space of the Analyzed GeneSet

  

Fig 3: CELL\_CORTEX(GO:0005938): Random ES distribution
  
Gene set null distribution of ES for
 **CELL\_CORTEX(GO:0005938)**
